# Comparison of molecular and immunocytochemical methods for detection of disseminated tumor cells in bone marrow from early breast cancer patients

**DOI:** 10.1186/1471-2407-14-514

**Published:** 2014-07-15

**Authors:** Bjørnar Gilje, Oddmund Nordgård, Kjersti Tjensvoll, Elin Borgen, Marit Synnestvedt, Rune Smaaland, Bjørn Naume

**Affiliations:** 1 Department of Hematology and Oncology, Stavanger University Hospital, Stavanger, Norway; 2 Laboratory for Molecular Biology, Stavanger University Hospital, Stavanger, Norway; 3 Division of Surgery and Cancer Medicine, Department of Pathology, Oslo University Hospital, Oslo, Norway; 4 Division of Surgery, Transplantation and Cancer Medicine, Department of Oncology, Oslo University Hospital, Oslo, Norway; 5 K.G. Jebsen Center for Breast Cancer Research, Institute for Clinical Medicine, University of Oslo, Oslo, Norway

**Keywords:** Disseminated tumor cells, RT-qPCR, Immunocytochemistry, Breast cancer, Bone marrow

## Abstract

**Background:**

Disseminated tumor cells (DTCs) have potential to predict the effect of adjuvant treatment. The purpose of this study was to compare two methods, reverse transcription quantitative PCR (RT-qPCR) and immunocytochemisty (ICC), for detecting breast cancer DTCs in bone marrow (BM) from early breast cancer patients.

**Methods:**

We investigated a subset (n = 313) of BM samples obtained from 271 early breast cancer patients in the “Secondary Adjuvant Taxotere Treatment” (SATT)-trial. All patients in this study had node positive or intermediate/high-risk node negative non-metastatic disease. The DTCs were detected by ICC using AE1-AE3 anti-cytokeratin monoclonal antibodies. Patients with DTCs detected in their BM by ICC after standard adjuvant fluorouracil, cyclophosphamide, epirubicin (FEC) chemotherapy were offered docetaxel treatment. For comparison, 5 × 10^6^ mononuclear cells from the aliquoted BM samples were also analyzed by RT-qPCR using a multimarker (MM) assay based on the tumor cell mRNA markers keratin 19 (*KRT19*), mammaglobin A (*hMAM*), and *TWIST1*. In the MM-assay, a sample was defined as positive for DTCs if at least one of the mRNA markers was positive.

**Results:**

The MM RT-qPCR assay identified DTCs in 124 (40%) of the 313 BM samples compared with 23/313 (7%) of the samples analyzed by ICC. The concordance between the MM RT-qPCR and ICC was 61% (Kappa value = 0.04) and twelve of the BM samples were positive by both methods. By RT-qPCR, 46/313 (15%) samples were positive for *KRT19*, 97/313 (31%) for *TWIST1*, and 3/313 (1%) for *hMAM* mRNA. There were no statistically significant associations between the individual mRNA markers.

**Conclusion:**

The RT-qPCR based method demonstrated more DTC-positive samples than ICC. The relatively low concordance of positive DTC-status between the two different assessment methods suggests that they may be complementary. The clinical relevance of the methods will be evaluated based on future clinical outcome data.

**Trial registration:**

ClinicalTrials.gov: NCT00248703.

## Background

Despite a continuous effort to improve cancer diagnostics and treatment, breast cancer remains a leading cause of death among women worldwide. Current adjuvant treatment decisions are dependent on well-known prognostic factors including TNM-staging and histological grade, as well as the estrogen receptor (ER), progesterone receptor (PgR), human epidermal growth factor receptor 2 (HER2), and more recently Ki-67-status [1]. The search for better prognostic factors, as well as predictors of the effect of adjuvant treatment, has led to a thorough evaluation of disseminated tumor cells (DTCs) and their persistence in bone marrow (BM) [2-5]. Moreover, DTCs have been shown to provide independent prognostic information in breast cancer patients [2-5]. However, more research is needed before the implementation of BM status in routine clinical practice. The predictive value of BM status as a tool in making adjuvant treatment decisions has yet to be investigated in randomized phase III trials. Furthermore, the detection of tumor cells in the BM does not always lead to disease relapse. Many patients with positive DTC status do not relapse, and DTCs can be detected in patients with ductal carcinoma in situ [6]. The mechanisms behind tumor dormancy and the possibility of tumor cell re-awakening are poorly understood. Interestingly, increasing evidence has emerged in the last few years supporting that the addition of bisphosphonates in the adjuvant treatment both reduces the risk of persistent DTCs and improves survival [7-10]. This supports the biological relevance of DTCs and the importance of methods to accurately assess the DTC-status when selecting patients for adjuvant treatment.

However, different methods are used to assess DTCs in the BM, and there is a clear need for standardization. Due to the very low frequency of DTCs in the BM, different methods are used to enrich tumor cells in the BM samples before detection. The enrichment can be based on density gradient centrifugation, flow cytometry, immunomagnetic beads, and membrane filtration [11]. Protocols based on immunocytochemistry (ICC) and reverse transcription quantitative PCR (RT-qPCR) are the most commonly used methods for DTC detection. When ICC is used for DTC detection, the results will be affected by the choice of keratin antibodies, as discrepancies between different antibody mixtures have been reported [11-13]. Similarly, the choice of mRNA markers, as well as different assays and platforms, affect the performance of RT-qPCR based DTC detection [4,14-19]. Thus, the comparison of studies based on different detection methods is challenging. Nevertheless, a few studies report the concordance between ICC-based and RT-qPCR-based DTC detection in breast cancer patients to be about 70-80% [20-22]; although, these numbers are primarily reflecting that the majority of patients have negative BM-status with both methodologies.

In the present study we compared a multimarker (MM) RT-qPCR assay, consisting of keratin 19 (KRT19), TWIST1, and mammaglobin A (hMAM), with ICC using the AE1-AE3 mAb for the detection of DTCs in 267 early breast cancer patients previously treated with adjuvant fluorouracil, cyclophosphamide, epirubicin (FEC) chemotherapy.

## Methods

### Patients

A total of 1121 patients were prospectively recruited to the “Secondary Adjuvant Taxotere Treatment” (SATT) trial from October 2003 to May 2008 [23]. In total, 313 BM samples from 271 of these patients were selected for the present study. All samples collected within a limited timeframe during the SATT trial were included in our study to avoid selection bias. Briefly, in the SATT-trial, only breast cancer patients with node positive or high-risk node negative disease (T1c/T2, GII-III, N0) were recruited. BM aspirations were performed twice in all patients. The first aspiration (BM1) was collected 8-12 weeks after standard adjuvant chemotherapy (FEC); whereas, a second BM aspiration was collected 6 months later (BM2). BM2-samples were analyzed by ICC for the presence of persisting DTCs after adjuvant chemotherapy. Patients with positive BM2 samples were then treated with 6 cycles of docetaxel every 3 weeks and two additional BM samples were collected from these patients approximately 1 month (BM3) and 13 months (BM4) after the last docetaxel infusion. Of the 313 BM samples included in our study, 92 were BM1, 187 were BM2, 14 were BM3, and 18 were BM4. In only a few cases, the BM-samples (BM1-4) were from the same patient, as all of our samples were collected consecutively during a limited timeframe. BM samples from 29 healthy women constituted the control group for the RT-qPCR analyses.

The SATT trial was approved by the Regional Committee for Medical and Health Research Ethics (REC South-East. Permit Number: S-03032) in compliance with the Declaration of Helsinki, and written consent was obtained from all patients. The study is registered in ClinicalTrials.gov (registration number NCT00248703, registration date November 3rd, 2005), and is reported according to the recommendations for tumor marker prognostic studies (REMARK) [24].

### BM sampling and handling

The BM samples were collected and processed as previously described [5]. Briefly, using local anesthesia, a small skin incision was first made to avoid contaminating epithelial cells before 5 ml of BM were aspirated from both posterior iliac crests using a syringe prefilled with 1 ml sodium-heparin. Mononuclear cells, including DTCs, were enriched from the BM aspirates by density centrifugation using Lymphoprep™ (Axis-Shield). The samples were then split into batches of 5 x 10^6^ cells for immediate preparation of cytospins (performed at Oslo University Hospital) and mRNA isolation (performed at Stavanger University Hospital). The remaining cells were stored in liquid N_2_ for later use.

### Immunocytochemistry

The cytospins were stained using the AE1-AE3 anti-cytokeratin antibodies as previously described [5,25]. The detection of DTCs was done by automated microscopy screening (Ariol SL50, Applied Imaging) or by manual screening with a light microscope. All candidate positive cells were reviewed by a pathologist (E.B.). Immunopositive cells were recorded according to recommended guidelines [5,25-28].

### RNA isolation and cDNA synthesis

Approximately 5 x 10^6^ cells were collected for RNA isolation. The mononuclear cell pellets were lysed in 350 μl RLT-lysis buffer (Qiagen) before total RNA was extracted using the RNeasy Mini Kit (Qiagen), according to the manufacturer’s protocol. All RNA samples were treated in a total volume of 10 μl with DNase I by incubating 1 μg total RNA from each sample with 1 unit RQ1 RNAse-free DNAse (Promega) in 1X First Strand Synthesis buffer (Invitrogen) containing 10 units RNAseOUT RNAse inhibitor (Invitrogen). The reaction mixture was incubated at 37°C for 30 min before the DNAse I was inactivated by adding 1 μl RQ1 stop solution, followed by incubation for 10 min at 65°C. Complementary DNA was synthesized by M-MLV reverse transcriptase in a total volume of 20 μl according to the manufacturer’s protocol (Invitrogen). Negative control samples without reverse transcriptase were included during cDNA synthesis.

### Real-time polymerase chain reaction assays

The amplification of KRT19 (GenBank Accession number NM_002276), hMAM (GenBank Accession number U33147), and TWIST1 (GenBank Accession number NM_000474) were performed as previously described, with minor modifications for the hMAM assay [4,18,29]. The concentration of the primers were reduced from 0.8 to 0.3 μM, and the amount of cDNA template increased to 50 ng in the hMAM RT-qPCR analysis to increase the sensitivity [4]. The quantification was performed in a LightCycler 480 (Roche Applied Science) instrument and the breakpoint cluster region (BCR: GenBank Accession number NM_004327) was used as a reference gene. KRT19 and TWIST1 were analyzed in duplicates; whereas, hMAM was analyzed in triplicates.

### Relative mRNA quantification

The mean Cq-values of the mRNA markers were normalized against the mean Cq-value of BCR and expressed relative to a calibrator sample (MDA-MB-361, Ambion Inc., Austin, TX) using the 2^ΔΔCq^ method [30]. BM samples from healthy controls were analyzed to determine the highest normal BM levels of KRT19 and TWIST1, which were then used as a cut-off for marker positivity. hMAM was not detected in the healthy control samples; therefore, any specific amplification in the patient samples was considered a positive result. If at least one of the mRNA markers (KRT19, hMAM, or TWIST1) included in the MM panel was positive, the patient was considered positive for DTCs.

### Statistics

The statistical analyses were performed using SPSS version 21.0 (http://www.spss.com). A two-sided p-value ≤0.05 was considered statistically significant. Missing data were excluded from the analyses. The concordance between the DTC-statuses assessed by RT-qPCR and ICC was calculated manually by dividing the number of concordant samples with the total number of analyzed samples, and by computing Kappa values [31]. The associations between categorical variables were analyzed by Fishers exact test for variables with two categories, and by the Linear-by-Linear Association test for variables with more than two categories.

## Results

We compared mRNA-based and ICC-based methods for analyzing the presence of DTCs in 313 BM samples from 271 breast cancer patients. The patients constituted a subgroup of the SATT-trial and the distribution of the clinicopathological parameters were similar to the entire SATT-trial [23]. The clinicopathological parameters and their relation to patients’ DTC statuses with both methods are shown in Table 1 for patients where BM samples were available 8-12 weeks (BM1) and/or 9 months (BM2) after FEC chemotherapy. No significant associations were found between clinicopathological parameters and BM-status, determined by ICC or the MM RT-qPCR assay.

**Table 1:** Clinicopathological data with ICC- and qPCR-status

	**All patients (n = 267)**		**ICC**			**qPCR**		
	**Number**	**(%)**	**Pos**	**Neg**	**p-value**	**Pos**	**Neg**	**p-value**
**Age (years)**					0.14			0.74
<55	220	(82.4)	21	199		91	129	
55-70	43	(16.1)	1	42		19	24	
Unknown	4	(1.5)						
**pT-status**					0.524			0.89
pT1a	5	(1.9)	2	3		1	4	
pT1b	14	(5.2)	0	14		6	8	
pT1c	115	(43.1)	7	108		52	63	
pT2	113	(42.3)	10	103		44	69	
pT3	14	(5.2)	3	11		7	7	
Unknown	6	(2.2)						
**pN-status**					0.14			0.88
pN0	109	(40.8)	7	102		44	65	
pN1	118	(44.2)	10	108		52	66	
pN2	27	(10.1)	4	23		10	17	
pN3	6	(2.2)	1	5		2	4	
Unknown	7	(2.6)						
**Histologic grade**					0.80			0.76
Grade I	18	(6.7)	0	18		7	11	
Grade II	151	(56.6)	16	135		63	88	
Grade III	90	(34.2)	6	84		38	52	
Unclassified	4	(1.5)	0	4		2	2	
Unknown	4	(1.5)						
**ER-status**					1.00			0.39
Positive	195	(73.0)	16	179		78	117	
Negative	67	(25.1)	5	62		31	36	
Unknown	5	(1.9)						
**PgR-status**					1.00			0.50
Positive	177	(66.3)	14	163		71	106	
Negative	84	(31.5)	7	77		38	46	
Unknown	6	(2.2)						
**HER2-status**					1.00			0.59
Positive	37	(13.9)	2	35		17	20	
Negative	204	(76.4)	16	188		83	121	
Unknown	26	(9.7)						

The ICC and RT-qPCR statuses were defined as positive if either BM1 or BM2 was positive. For 238 patients, either BM1 or BM2 was available; whereas, both BM1 and BM2 were available for 29 patients. BM3 and BM4 results were excluded from this analysis because they were only analyzed if ICC BM2 was positive. Four of the 271 patients had only BM3 or BM4 available and were excluded from the analysis in this table.

**Figure 1:**
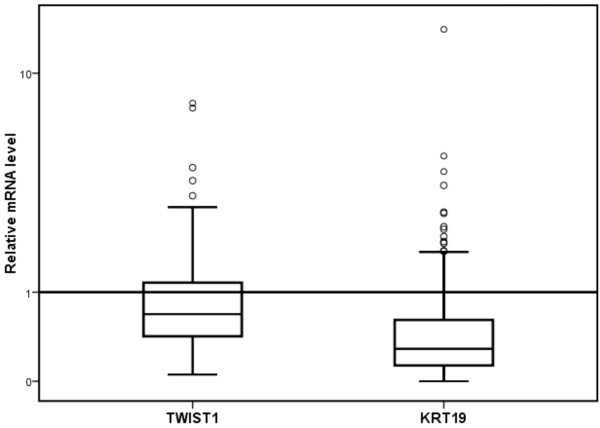
**Relative levels of TWIST1 and KRT19 mRNA in BM samples from 267 early breast cancer patients.** The levels were calculated using the 2^ΔΔCq^ method and normalized by dividing by the highest level in the control samples. The horizontal line represents the highest level in the control samples with the relative value of one. hMAM is not shown in the plot because no expression was found in the normal control samples.

The BM DTC-status was positive in 124/313 (40%) samples by our MM RT-qPCR assay as compared to 23/313 (7%) samples by ICC. Among the 124 MM-positive samples, 46 (37%) were positive for KRT19, 97 (78%) for TWIST1, and 3 (2.4%) for hMAM. In addition, TWIST1 was positive in 19 of the 46 KRT19 positive samples. No significant association was found between the separate mRNA markers. The relative BM levels of the markers in the 313 samples from early breast cancer patients are shown in Figure 1. The comparison between ICC and the separate mRNA markers/MM panel is summarized in Table 2. Of the 313 samples analyzed, 190 (61%) showed concordance between the MM RT-qPCR assay and ICC (Kappa value 0.045). Only 12 samples were positive by both methods, but 135 samples were positive by at least one method. About 57% of the samples were negative by both methods. The concordances between the individual mRNA markers and ICC were 81% for KRT19, 67% for TWIST1, and 93% for hMAM.

**Table 2:** Concordance between ICC and mRNA markers

		**ICC**		**Concordance**	**Kappa**
		**Pos**	**Neg**		**Value**
Multimarker	Pos	12	112		
	Neg	11	178	0.61	0.045
KRT19	Pos	4	42		
	Neg	19	248	0.81	0.020
hMAM	Pos	2	1		
	Neg	21	289	0.93	0.14
TWIST1	Pos	9	88		
	Neg	14	202	0.67	0.035

The DTC detection results at various sampling time points are shown in Table 3. In BM1, 47.8% of the samples were positive for DTCs by the MM RT-qPCR assay as compared to 33.7% in BM2. The corresponding ICC results were 7.6% and 5.9%, respectively. Thus, by both methods, fewer patients had DTCs in BM2 compared with BM1. For all BM1-4 samples, the number of positives was much higher, on average 5-fold, by MM RT-qPCR than ICC. It is important to note that BM3 and BM4 have a higher frequency of positive samples because these samples were only collected from patients with a positive BM2 sample.

**Table 3:** Distribution of BM1-4 with ICC and qPCR data

**BM number**	**Total**	**ICC**		**qPCR**		**Concordant**
	**313**	**Positive (%)**		**Positive (%)**		**BM results (%)**
BM 1	92	7	(7.6)	44	(47.8)	(53)
BM 2	187	11	(5.9)	63	(33.7)	(66)
BM 3	14	2	(14.3)	10	(71.4)	(43)
BM 4	18	3	(16.7)	7	(38.9)	(56)

## Discussion

This study was undertaken to compare ICC with a MM RT-qPCR assay for the detection of DTCs in BM after adjuvant chemotherapy in early breast cancer patients. Our study revealed a markedly higher frequency of positive samples by both the MM RT-qPCR assay and the individual mRNA-assays compared with ICC. Multiple mRNA markers clearly contributed to a higher number of positive samples compared to only using single markers. The relatively high (61%) concordance between ICC and RT-qPCR is primarily because a large fraction of the samples were negative by both methods. Accordingly, the kappa observer agreement value was only 0.04, suggesting that the apparent concordance was primarily due to chance. In principle, the ICC assay should stain, among others, KRT19 positive cells. Thus, we expected better concordance between the ICC and the KRT19 mRNA results. However, only 4 out of 313 samples were positive by both methods and as many as 42 ICC-negative samples were positive for KRT19 mRNA. One possible explanation for this is that the KRT19 mRNA assay is more sensitive than the ICC assay. The 19 ICC-positive samples that were not detected by the KRT19 mRNA assay might be explained by detection of KRT19-negative DTCs that express other keratins detected by the ICC approach.

The low concentration of DTCs in BM samples may affect reproducibility in both detection methods. Many samples had levels near the detection limit for KRT19 mRNA; whereas, the ICC-assay was able to detect only a single cell in the majority of positive samples. It follows from the Poisson distribution of rare events that there is a roughly 35% risk that a second sample would be a false negative. Hence, the reproducibility of DTC detection might be enhanced by analyzing larger sample volumes. On the other extreme, it was recently shown that screening a very large volume of peripheral blood by leukapheresis revealed DTCs in 90% of non-metastatic breast cancer patients [32]. Such high numbers of DTCs does not correlate with the risk of relapse for this patient group, and thus implies a dramatic increase in detection of clinically irrelevant cells.

The hMAM mRNA assay was only positive in a very small number of samples (3/313); therefore, it might be of limited value in combination with KRT19 mRNA in the post-adjuvant treatment setting. Indeed, 2 of the 3 positive hMAM samples were also positive for KRT19 mRNA and by ICC, with convincing DTC-counts of 2 and 46 by ICC. The remaining hMAM positive sample was TWIST1-positive and KRT19- and ICC-negative. Thus, it seems that hMAM contributes to the identification of a very small subgroup of patients, possibly those with a very high risk, consistent with our previous report on hMAM [4,18].

TWIST1 was shown to add prognostic information to a DTC MM panel described by Tjensvoll et al. [4]. Interestingly, we noted that a substantially higher number of patients had elevated TWIST1 mRNA levels in our present study [4]. The clinical follow-up will ultimately help determine the relevance of this discrepancy. As TWIST1 is a proposed epithelial-mesenchymal-transition (EMT)-marker [33], the higher number of positive samples might indicate that a substantial portion of patients have DTCs not expressing keratins [34]. The number of TWIST1 positive samples, however, exceeds the anticipated number of clinical relapses. Thus, our assay might be too sensitive, or the cut-off level needs refinement to reveal only clinically relevant information. ROC analysis in relation to clinical outcome data, when available, may reveal an optimal cut-off value. However, this will require confirmation in a validation cohort.

The high number of DTC positive samples by the RT-qPCR approach is in part explained by the high number of TWIST1 positive samples. In later years, there has been much focus on mesenchymal markers to detect cells that have undergone EMT as part of the metastatic process. This is thought to be a reversible process in which the cancer cells gain mesenchymal properties to be able to infiltrate different tissues and give rise to micro- and ultimately macro metastases [34]. Yu et al. showed that in circulating tumor cells a shift towards higher expression of EMT-markers is associated with tumor progression [35]. Thus, we might speculate whether cells transiently expressing mesenchymal genes, like TWIST1, comprise the subgroup of DTCs with stem-cell properties and, therefore, the proportion of DTCs that harbor metastasis-generating abilities [36]. The loss of epithelial characteristics may imply that these cells are difficult to detect by most commonly used ICC DTC assays.

The discrepancy between the RT-qPCR based and the ICC-based DTC detection is not surprising based on previous studies. Becker et al. found agreement between ICC (with the A45-B/B3 mAb) and KRT19 mRNA detection in 73% of the 385 cases, in line with our KRT19 qPCR results (81% agreement). Although, the results are biased since the majority of patients were negative by both methods. In fact, a kappa value of 0.39 can be computed based on their reported data, confirming this suspicion to some extent. Moreover, they demonstrated a 35% positive rate for both ICC and KRT19 mRNA and 49% of the patients were positive by at least one of the methods. The time of BM-collection might be an important difference between their study and the present one. We collected BM after adjuvant chemotherapy; whereas, Becker et al. collected the majority of samples prior to surgery and only a few (n = 63) after surgery and chemotherapy. This may have contributed to the much lower number of positive samples based on both ICC and KRT19 mRNA in our study. Others have reported concordance in the same range as in our study. Benoy et al. reported concordant results in 75% of the samples; whereas, Slade et al. found agreement between the methods in 71% of the samples [21,22]. Molloy et al. compared a MM RT-qPCR assay with ICC in a large population of 733 patients and found both to be significantly predictive of poorer outcome. However, the RT-qPCR assay was applied to blood samples (circulating tumor cells) and the ICC to BM samples (DTCs). Thus, a direct comparison with the current study is difficult because the samples were collected from different body regions in addition to being analyzed by two different methods [37].

A general issue regarding mRNA-based DTC detection is the background level of epithelial transcripts in white blood cells. However, comparison with blood samples from a normal control cohort may compensate for this issue, allowing threshold values for pathological marker levels in blood to be established. The latter strategy was utilized in the current study to minimize the number of false positives due to such background expression in leukocytes.

Despite clear evidence that DTCs in BM in early breast cancer patients predict a poor outcome, a better understanding is needed for these analyses to be implemented in the routine clinical management of patients. Braun et al. found, in their large pooled analysis of 4703 patients, a significant prognostic value of BM DTC-status in all patients including the lymph-node negative subgroup [3]. On the other hand, several smaller studies, e.g. by Langer et al., did not find any significant DTC-specific difference in overall and breast cancer specific survival in 411 clinically lymph node negative patients, a result that may be caused by the low number of patients in the study [3,38]. However, this result emphasizes that the prognostic value of BM DTC-status might be strongest in already defined high-risk patients. In the current analysis, only intermediate/high-risk patients, i.e. patients with higher-risk node negative or node positive disease, were included. Thus, this may be a group where the BM-DTC status may add clinically relevant prognostic information.

Few studies have investigated the impact of BM-DTCs after initial therapy. In a pooled analysis, Janni et al. demonstrated that DTCs can be detected several years after diagnosis [39]. The persistence of BM DTCs after neoadjuvant treatment was also associated with worse prognosis in a recent study [40], and our previous results showed reduced survival for patients with persistent DTCs, assessed by our mRNA MM-assay (hMAM, KRT19, and TWIST1), after surgery [41]. The majority of studies so far have used ICC for the detection of DTCs in the BM; although, some studies also used RT-qPCR. The DTC-detection by ICC is largely based on pan-cytokeratin antibodies, but different antibody combinations have also been used with varying results. Effenberger and colleagues found that ICC detection and prognostic relevance were different for the two most commonly used pan-cytokeratin antibody combinations (A45-B/B3 (A45) and AE1-AE3 (AE)) [12]. The AE mAb was more prognostic for the lymph node positive patients. Accordingly, the AE mAbs were utilized in the current SATT-trial, consistent with the inclusion of a higher risk population.

## Conclusions

In conclusion, this study is to our knowledge the largest comparison between ICC- and RT-qPCR-based DTC detection methods in BM samples collected after adjuvant chemotherapy in a defined high-risk early breast cancer population. We detected more positive BM samples with RT-qPCR assays, based on KRT19, hMAM, and TWIST1 mRNAs, than with ICC. The clinical implications of these findings, however, await future clinical follow-up. Due to a potential shift in DTC phenotype, we included the mesenchymal TWIST1 mRNA marker in an attempt to detect the subpopulation of DTCs lacking epithelial characteristics. Hopefully, this might help identify additional patients with clinically relevant DTCs. The current findings support that the different means of detection could be complementary and that both RT-qPCR and ICC should be further studied as methods for DTC detection in early breast cancer patients.

## Abbreviations

DTC: Disseminated tumor cell; BM: Bone marrow; SATT: Secondary Adjuvant Taxotere Treatment; ICC: Immunocytochemistry; FEC: Fluorouracil, epirubicin, cyclophosphamide; MM,: Multimarker; KRT19: Keratin 19; hMAM: Mammaglobin A; RT-qPCR: Reverse transcription quantitative polymerase chain reaction; TNM: Standard tumor-node-metastasis classification according to AJCC/UICC 2002; ER: Estrogen receptor; PgR: Progesterone receptor; HER2: Human epidermal growth factor receptor 2; BCR: Breakpoint cluster region; EMT: Epithelial-mesenchymal-transition.

## Competing interests

The authors declare that they have no competing interests.

## Authors’ contributions

BG, ON, KT, RS, and BN drafted the manuscript. BG, BN, RS, MS and ON were responsible for the study design. BG and ON performed the data analysis and carried out the statistics. EB performed immunocytochemistry detection of DTCs and BG performed the RT-qPCR-based detection of DTCs. All authors read and approved the final manuscript.

## Pre-publication history

The pre-publication history for this paper can be accessed here:

http://www.biomedcentral.com/1471-2407/14/514/prepub
